# Serum Anti-Thyroglobulin Autoantibodies Are Specific in Predicting the Presence of Papillary-like Nuclear Features and Lymphocytic Infiltrate in the Thyroid Gland

**DOI:** 10.3390/diagnostics13122042

**Published:** 2023-06-13

**Authors:** Daniela Cabibi, Antonino Giulio Giannone, Sandro Bellavia, Roberta Lo Coco, Anna Lo Bianco, Eleonora Formisano, Gregorio Scerrino, Giuseppa Graceffa

**Affiliations:** 1 Unit of Anatomic Pathology, Department of Health Promotion Mother and Child Care Internal Medicine and Medical Specialties (PROMISE), University Hospital AOU Policlinico “P. Giaccone”, University of Palermo, 90127 Palermo, Italy; 2 Unit of General and Emergency Surgery, Department of Surgical Oncological and Stomatological Sciences (DICHIRONS), University Hospital AOU Policlinico “P. Giaccone”, University of Palermo, 90127 Palermo, Italy; 3 Unit of General and Oncological Surgery, Department of Surgical Oncological and Stomatological Sciences (DICHIRONS), University Hospital AOU Policlinico “P. Giaccone”, University of Palermo, 90127 Palermo, Italy

**Keywords:** anti-Thyroglobulin, anti-Tg antibodies, anti-TPO, thyroid papillary-like nuclear features, thyroid lymphocytic infiltrates, thyroid carcinogenesis

## Abstract

(1) Background: Previous studies have reported a correlation between serum anti-Thyroglobulin-antibodies (TgAb) and papillary thyroid carcinoma. The aim of our study was to evaluate whether serum TgAb and anti-thyroid-peroxidase antibody (TPO) positivity was also related to pre-neoplastic histological changes such as papillary-like nuclear features (PLNF) and with the presence of lymphocytic infiltrate (LI) in thyroid surgical specimens. (2) Methods: The study was retrospectively carried out on 70 consecutively recruited patients who underwent thyroidectomy for benign process and whose TgAb and TPOAb values were retrieved from clinical records. Histological sections of thyroid surgical samples were revised, looking for PLNF and lymphocytic infiltrate. HBME1 expression was assessed by immunohistochemistry. (3) Results: Our results showed a significant association between TgAb, PLNF, and lymphocytic infiltrate. The presence of TgAb was highly specific, but less sensitive, in predicting the presence of PLNF (sensitivity = 0.6, specificity = 0.9; positive predictive value (PPV) = 0.88; negative predictive value (NPV) = 0.63). TgAb positivity showed a good association with the presence of lymphocytic infiltrate (sensitivity = 0.62, specificity = 0.9; PPV = 0.88 and NPV = 0.68). HBME1 immunoreactivity was observed in the colloid of follicles showing PLNF and/or closely associated with LI. (4) Conclusions: The presence of PLNF and LI is associated with serum TgAb positivity. The presence of TgAb and of LI could be triggered by an altered thyroglobulin contained in the HBME1-positive colloid, and could be a first defense mechanism against PLNF that probably represent early dysplastic changes in thyrocytes.

## 1. Introduction

The diagnosis of papillary thyroid carcinoma is based on the presence of a nodule with papillary or follicular architecture and widespread characteristic “papillary” nuclear features, such as nuclei with chromatin clearing, grooves, nuclear enlargement, and nuclear membrane irregularity [1,2]. Focal and poorly represented nuclear features of papillary carcinoma do not configure the picture of the overt malignant neoplasm and can be present in different contexts, such as in multinodular goiters and in autoimmune thyroid diseases, especially in Hashimoto’s thyroiditis. In the latter setting, some authors considered these changes as “pre-neoplastic changes”, reporting them as “follicular epithelial dysplasia showing papillary thyroid carcinoma-like nuclear alterations” [3,4], but whether these features are truly pre-neoplastic changes remains a controversial point. Moreover, “papillary-like nuclear features” (PLNF) are the typical changes described in cases of “non-invasive follicular thyroid neoplasm with papillary-like nuclear features” (NIFTP) and of “encapsulated follicular variant of papillary thyroid carcinoma” (EFVPTC) [5].

The presence of anti-Thyroglobulin serum autoantibodies (TgAb) has been reported as an independent risk factor for papillary thyroid carcinoma (PTC), with increase in TgAb prevalence in subjects with PTC and with influence on the prognosis [6,7,8,9].

TgAb are endogenous molecules that recognize specific epitopes of Thyroglobulin (Tg) with specificity and variable affinity. These molecules are mainly polyclonal, of IgG type, and rarely (1%) IgA or IgM type [10].

They are linked to the autoimmune diseases and/or the differentiated thyroid cancer. Recently, it has been hypothesized that the relationship between TgAb and PTC does occur in an earlier phase than the stage of frank carcinogenesis [11].

Thyroglobulin is an intrathyroidal protein involved in the synthesis and storage of thyroid hormones. It is a homodimeric glycoprotein formed by two polypeptide chains with 20 potential linked glycosylation sites, of which 16 sites are glycosylated in the mature protein. Thyroglobulin undergoes several post-translational modifications, such as glycosylation, sulfation, or phosphorylation, that are partly responsible for the high glycoprotein antigenicity. Iodination in position 117–132, 304–318, and 1931–1945 of the polypeptide chains promotes the antigenic effect of Tg. The absence of iodination of peptides located in position 179–194, 2529–2545, and 2540–2554 consolidates the antigenic effect of Tg [12,13,14]. Iodine may increase the post-translational modification of Tg, enhancing the immunopathogenicity, leading to thyroiditis induction. Furthermore, iodine may produce apoptotic/necrotic effects on thyrocytes, initiating the presentation of thyroid antigens and triggering autoimmunity phenomena [13].

Disorders of Tg post-translational modifications (e.g., glycosylation, sialylation) lead to impaired functioning of Tg and play a pathological role in chronic thyroid diseases such as cancer and autoimmunity [15,16]. Thyroid carcinogenesis and thyroid autoimmunity are linked to changes in sialylation and fucosylation. Decreased content of sialic acid in Tg glycans was observed in patients with thyroid cancer and Graves’s disease [17,18,19]. Thus, the same mechanisms may be involved in carcinogenic processes or in autoimmune processes.

Noteworthily, many studies suggested the existence of a relationship between the presence of lymphocytic infiltrate and the presence of more differentiated papillary carcinomas with a better prognosis, suggesting a protective role of the lymphocytic infiltrate against thyroid neoplasms [20,21,22].

The immunohistochemical overexpression of Hector Battifora Mesothelial-1 antigen (HBME-1) is detected in thyroid malignant neoplasms, with a high sensitivity and specificity for papillary thyroid carcinoma, with this immunomarker virtually absent in normal thyroid tissue and rarely positive in benign lesions of the thyroid gland [23,24,25]. Thyroid papillary carcinoma usually shows a striking membranous staining with this antibody.

The immunohistochemical utility of several markers has been reported in well-differentiated thyroid neoplasms. In particular, the combination of HBME1, Galectin-3, and CK19 positivity, together with CD56 negativity, represent the most useful panel used by pathologists to discriminate between benign and malignant neoplasm, especially when a follicular proliferation with an equivocal morphology is encountered.

However, HBME-1 is a marker commonly used alone in the approach to thyroid neoplasms as one of the best tools to screen thyroid malignancy, because the diagnostic utility of CK19 and Galectin-3 in papillary thyroid carcinoma is controversial. Since these markers are also observed in several thyroid benign diseases, such as goiter, Hashimoto thyroiditis, follicular adenomas, etc., they appear less specific than HBME1. Despite many attempts to hunt the cryptic target antigen of HBME1, its identity still remains unclear in thyroid neoplastic cells [26].

To our knowledge, no studies have been performed to assess if a relation does exist between the presence of serum autoantibodies and the presence of lymphocytic infiltrate in the thyroid gland. The aim of this study was to assess the relationship between serum positivity for TgAb, anti-thyroperoxidase antibodies (TPO), the presence of lymphocytic infiltrate, and the presence of PLNF on surgical samples of thyroid gland surgically resected for non-neoplastic pathology. As the presence of morphological changes in thyrocytes could lead to the production of an altered thyroglobulin protein, with antigenic effects, we hypothesized a relationship between the presence of histological alterations in the thyroid gland, the presence of inflammatory infiltrate, and the presence of serum TgAb elevation. Furthermore, we investigated the existence of a relationship between serum TgAb, lymphocytic infiltrates, and HBME-1 immunoreactivity in thyroid surgical specimens.

## 2. Materials and Methods

The study was retrospectively carried out on a casuistry of 70 patients, 24 men and 46 women, with a mean age of 52 years (range of age 22–76 years). The patients underwent total thyroidectomy or hemithyroidectomy in the period between 2015 and 2020, at the Unit of General and Oncological Surgery and the Unit of General and Emergency Surgery of the University Hospital “Paolo Giaccone” Department of Surgical Oncological and Stomatological Sciences (DICHIRONS), University of Palermo.

All patients had undergone pre-operative serological dosing of TgAb and TPOAb by peripheral venous blood sampling and their values were retrospectively retrieved from the corresponding medical records. Cases lacking serological TPOAb and TgAb laboratory results were excluded from the study.

Cut-off values >34 UI/mL and >20 UI/mL were used to define serum positivity for TPOAb and TgAb, respectively.

Hematoxylin and eosin (H&E)-stained sections, obtained from the formalin-fixed paraffin-embedded (FFPE) surgical specimens archived at the Unit of Anatomic Pathology of the University Hospital “Paolo Giaccone”, Department of Health Promotion, Mother and Child Care, Internal Medicine and Medical Specialties (PROMISE), University of Palermo, were retrospectively reviewed to assess the presence of lymphocytic infiltrate and the presence of PLNF. The cases were pre-viewed by two resident pathologists (SB, RLC). Finally, all histological and immunohistochemical features were assessed by two expert pathologists (DC, AGG), with high concordance among the pathologists (k index of 0.88). The most controversial cases were collegially reviewed and discussed to achieve the best classification.

Immunohistochemical stains were carried out with a BenchMark ULTRA automated slide staining system (Ventana Medical Systems, Tucson, AZ, USA) according to the manufacturer’s instructions, using the primary antibody antiHBME-1 (mouse monoclonal, clone HBME1, Cell Marque, Rocklin, CA, USA). For the immunohistochemical stains, the 3,3-diaminobenzidine kit was used as chromogen. The slides were observed on a Leica DM2000 microscope (Leica Microsystems, Wetzlar, Germany). The microphotographs were obtained using a Leica DFC320 camera (Leica Microsystems, Wetzlar, Germany).

Papillary-like nuclear features were evaluated as absent/present on H–E-stained sections; they were registered as present when nuclear clearing and/or grooves were present in isolated foci of thyrocytes within a goiter or a thyroiditis and when present in the context of nodules referable to “Non-Invasive Follicular Thyroid Neoplasm with Papillary-Like Nuclear Features” (NIFTP). The assessment of papillary-like nuclear features was carried out referring to the World Health Organization Classification of Endocrine Tumors criteria, represented by size and shape (enlargement, overlapping, crowding, and elongation), membrane irregularities (irregular contours, grooves, folds, and pseudoinclusions), and chromatin characteristics (clearing with margination, glassy nuclei). For the cases categorized as having PLNF, nuclear features from at least two of the three above-mentioned categories were identified to a substantial degree [27].

HBME1 immunostaining was evaluated as a “membranous pattern” when the cytoplasmic membrane of thyrocytes was stained. HBME1 “colloidal pattern” was defined when only the colloid within the follicle was stained, without a concurrent membranous staining.

The statistical analysis was carried out using the Yates Chi-square test and the Fisher test. Statistical significance was assumed for *p* values < 0.05. The sensitivity, specificity, positive predictive value (PPV), and negative predictive value (NPV) of TgAb and TPOAb with respect to the presence of PLNF and of lymphocytic infiltrate were also calculated.

## 3. Results

The case series consisted of 70 patients whose underwent total or partial surgical resection of thyroid gland with diagnosis of benign processes: 2 NIFTP, 2 Graves’s disease, 9 follicular adenomas, 44 multinodular goiters, and 12 chronic lymphocytic thyroiditis.

Both TgAb and TPOAb were present in 19 patients (2/2 with NIFTP, 6/10 with follicular adenomas, 6/44 with multinodular goiter, 1/2 with Graves’s disease, and 8/12 with chronic lymphocytic thyroiditis. In addition, seven patients showed only serum TgAb positivity and six patients only serum TPOAb positivity. Finally, both antibodies were absent in 38 patients. Table 1 summarizes the results on the presence of serum antibodies in each disease type.

The patients were divided into two groups on the basis of the serological TgAb and TPOAb findings.

Group 1: TgAb-positive (TgAb+) patients = 26/70 (37%), of which 19/26 patients (73%) were also TPOAb+, and the remaining 7/26 patients (26.9%) were TPOAb-.

Group 2: TgAb-negative (TgAb-) patients = 44/70 (63%), of which 38/44 (86.4%) patients were also TPOAb−, and 6/44 (13.6%) patients were TPOAb+.

**Figure 1:**
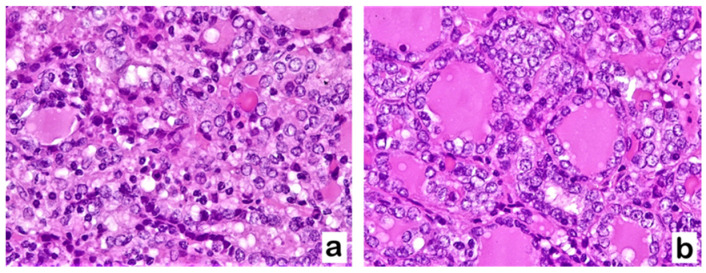
TgAb+/TPOAb− patient with goiter (**a**) and TgAb+/TPOAb− patient with chronic lymphocytic thyroiditis (**b**) showing PLNF: thyrocytes are characterized by nuclear enlargement and crowding, nuclear membrane irregularities with grooves, and nuclear clearing with glassy appearance (Hematoxylin–Eosin staining, (**a**,**b**): 400×).

The histological examination showed in 34/70 cases (48.6%) the presence of both PLNF and lymphocytic infiltrate. In 28/70 cases (40%), both alterations were absent. PLNF, consisting of alterations of enlarged nuclei with clear chromatin, grooves, and nuclear membrane irregularities are shown in Figure 1.

### 3.1. Relationship between PLNF, Lymphocytic Infiltrate, and TgAb

**Figure 2:**
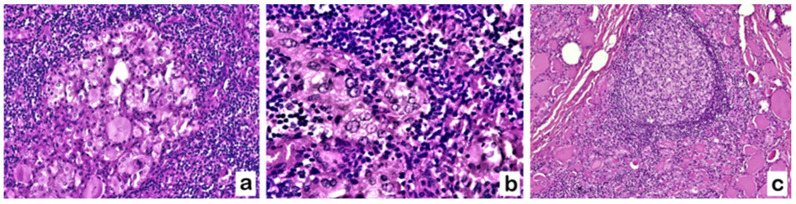
TgAb+/TPOAb− patient with goiter: thyrocytes with PLNF associated with lymphocytic infiltrate, sometimes with secondary lymphoid follicles with germinal center. (Hematoxylin–Eosin staining. (**a**,**b**): 200×; (**c**): 100×).

The presence of both PLNF and lymphocytic infiltrate was detected in 21/26 TgAb+ patients (80.8%) and only in 13/44 TgAb− patients (29.5%). Lymphocytic infiltrate was evidenced close to clusters of follicles showing PLNF. It consisted of small, tightly packed lymphocytes, as shown in Figure 2a,b. Germinal centers were sometimes visible [Figure 2c].

In 27/70 cases lacking both PLNF and lymphocytic infiltrate, the serum levels of TgAb were under the cut-off, so these cases were classified as TgAb-negative cases. The presence of PLNF, isolated or together with the lymphocytic infiltrate, was observed in 39/70 cases (55.7%). Among them, 23/26 (88.5%) were TgAb+, while only 16/44 (36.4%) were TgAb−.

The total number of cases showing the presence of lymphocytic infiltrate, isolated or associated with the PLNF, was 37/70 (52.86%) cases. Among the cases showing PLNF, 23/26 (88.5%) were TgAb+ and only 14/44 were TgAb− (31.8%). In conclusion, 23/26 TgAb+ cases showed the presence of at least one of the two alterations, (PLNF and/or lymphocytic infiltrate); only one case lacking both alterations was positive for TgAb (1/26). The results are reported in Table 2.

Yates’s Chi-square test showed a significant statistical difference regarding the presence of PLNF and lymphocytic infiltrate between group 1 (TgAb+) and group 2 (TgAb−), with *p*-value < 0.05.

### 3.2. Relationship between PLNF, Lymphocytic Infiltrate, and the Presence of TgAb in TPOAb+ and TPOAb− Patients

In 17/19 (89.5%) TgAb+/TPOAb+ patients, we observed both PLNF and lymphocytic infiltrate. The remaining cases had at least one of the two alterations. PLNF were present in 6/7 (85.7%) TgAb+/TPOAb− cases and in 16/38 (42.1%) TgAb−/TPOAb− cases. The presence of a lymphocytic infiltrate was observed in 5/7 (71.4%) TgAb+/TPOAb− cases and in 10/38 (26.3%) TgAb−/TPOAb− cases (Table 3).

Statistical analysis did not show a significant difference (*p* > 0.05).

### 3.3. HBME1 Results

The HBME1 membranous immunohistochemical pattern, as usually seen in papillary carcinoma, has never been found in the cases of the present study.

**Figure 3:**
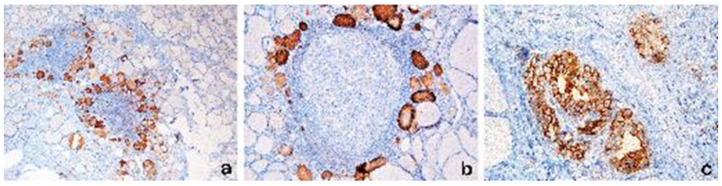
(**a**,**b**): HBME1 colloidal pattern of immunoreactivity restricted to the colloid of follicles with PLNF changes, associated with lymphocytic infiltrate. The HBME1 immunoreactivity pattern is different from the membranous HBME1 pattern seen in thyrocytes of PTC, which is shown for comparison in (**c**). (HBME1 immunostaining, (**a**): 40×; (**b**): 200× (**c**): 400×).

Instead, in the great majority of the cases (21/23, 91.3%) we observed the presence of a “colloidal pattern” on HBME1 staining, consisting of colloid immunoreactivity within the follicles, with thyreocytes showing PLNF and/or closely associated with LI [Figure 3a,b]. The colloidal pattern of HBME1 reactivity was rarely observed in cases negative for PLNF and LI. In Figure 3c, the membranous pattern of a papillary carcinoma is illustrated for comparison.

## 4. Discussion

The role of serum TgAb has been investigated by many studies [11,28,29,30,31,32,33,34]. TgAb have been considered an independent risk factor for papillary carcinoma. A positive correlation between the serum positivity for TgAb and the presence of papillary carcinoma has been evidenced. Moreover, in patients with pre-operative serum TgAb positivity, an unfavorable prognostic role of TgAb persistence in the post-operative period has been reported. The TgAb decline after total thyroidectomy and persistent/increasing levels may indicate a higher risk of cancer persistence/recurrence [29].

Durante et al. reported that PTC patients with positive postoperative serum TgAb titers are more likely to have persistent/recurrent disease than similar patients who are consistently TgAb-negative, while the disappearance of TgAb titers within the first postoperative year seems to be associated with a more favorable prognosis [9]. Recently, TgAb levels have been associated with malignant risk also for follicular carcinoma, and they may be useful markers for preoperative differential diagnosis of follicular neoplasms [30]. Adhami et al. demonstrated that elevated serum TgAb levels and TSH > 1 mIU/L were independently and synergistically associated with an increased risk of thyroid carcinoma in patients with indeterminate results at fine-needle aspiration cytology (FNAC) [31].

Our study highlights a statistically significant relation between the presence of serum TgAb and the presence of PLNF and/or lymphocytic infiltrate in surgically resected non-neoplastic thyroid gland. The presence of serum TgAb is highly specific, although not very sensitive, in predicting the presence of PLNF (specificity = 0.9, sensitivity = 0.6, positive predictive value (PPV) = 0.88, negative predictive value (VPN) = 0.63) and in predicting the presence of lymphocytic infiltrate (specificity = 0.9, sensitivity = 0.62, PPV = 0.88, VPN = 0.68).

Conversely, TPOAb positivity does not show a statistically significant correlation with the presence of PLNF and lymphocytic infiltrate (*p* > 0.05, Table 2). Therefore, serum TgAb positivity appears highly specific in predicting the presence of PLNF and lymphocytic infiltrate, independently from TPOAb status.

The co-existence of both types of autoantibodies is strongly indicative of an autoimmune pathology such as Hashimoto’s thyroiditis. Almost all Hashimoto’s thyroiditis patients and nearly 75% of Graves’s disease patients have detectable TPOAbs; on the contrary, the presence of TgAb alone is not sufficient for the diagnosis of an autoimmune process. In fact, TgAbs are detectable in 25% of thyroid cancer patients and in 10% of the general population [32,33], where the meaning is not clear.

To better understand the findings of our study, we further investigated the immunohistochemical expression of HBME-1, a commonly used marker in the approach to thyroid neoplasms. The first data about HBME1 in differentiated thyroid carcinomas were published in 1996, when the combined application of HBME-1 and CD15 was proposed to highlight cellular glycoconjugates changes related to malignant transformation [34]. To date, the identity of the target antigen of HBME1 still remains unclear [26].

HBME-1 over-expression has been reported in thyroid carcinomas with 92.8% sensitivity [35], and it is absent in nonneoplastic diseases and in normal thyroid tissue [36,37]. Considering the high specificity of this marker, some studies stressed the need for active surveillance of benign cases showing focal HBME-1 staining, for their possible degeneration towards an incipient malignant neoplasm [38].

Noteworthily, HBME1 expression “can be present in focal areas of Hashimoto’s thyroiditis and the cells in these areas may show nuclear features of papillary thyroid carcinoma. HBME1 positivity per se should not be equated with a diagnosis of papillary thyroid carcinoma in this setting” [38]. In PTC, HBME-1 typically shows a membranous pattern of immunoreactivity in the neoplastic follicular cells.

In our study, as expected, we did not observe a membranous HBME1 pattern. However, cases with PLNF and lymphocytic infiltrates, even if they were not clearly malignant, showed a peculiar HBME-1-positive immunostaining, limited to the colloidal substance of follicles with PLNF, surrounded by lymphocytic infiltrates.

From a practical point of view, this different reactivity pattern of HMBE1 at immunohistochemistry may allow the pathologist to differentiate malignancy in cases with previous indeterminate cytology specimens. We think that this “colloidal” staining pattern is due to the altered composition of Tg, leading to abnormal immunogenic activity attracting lymphocytes and simultaneously making it capable of reacting with HBME1.

This altered Tg could act as “neoantigen”, with recruitment of inflammatory elements such as T-lymphocytes. This may be facilitated by some polymorphisms that can induce the activity of T-cells [39]. Moreover, it could be a stimulus to produce TgAbs. Several alterations of the Tg gene linked to neoplastic transformation have been reported [40]. Mutations in the Tg gene have been identified that interfere with several crucial pathways in cell cycle regulation, including the RAS–MAP kinase pathway [41]. Furthermore, a new functional microRNA was discovered encoding within the Tg gene, whose downregulation leads to the deregulation of the pathways related to MAP kinase signaling [42].

The normal functions and dynamics of Tg require specific post-translational modifications. These are multistep enzymatic processes occurring in the endoplasmic reticulum and Golgi apparatus that change the properties of a protein by adding a modifying group, such as glycosylation and sialylation. The glycosylation of Tg is necessary for the proper functioning and immunoreactivity of Tg [43,44]. Moreover, the neoplastic transformation of thyroid follicular cells to PTC and follicular thyroid carcinoma (FTC) is associated with an increase in sialylation [19,45,46]. Glycosylation is also the main processes affected in thyroid autoimmunity [19] and could represent the “trait d’union” to understand the processes leading to coexistence of serum TgAb, lymphocytic infiltrates, and PLNF in non-neoplastic thyroid gland, as evidenced in our study.

Consistent with the first studies about HBME1, stating that “HBME1 highlights cellular glycoconjugates changes related to malignant transformation” [34], we hypothesize that an important role could be played by anomalous Tg glycosylation, which leads to the formation of the HBME1 target antigen, determining the immunoreactivity for HBME1 of the colloidal substance and simultaneously acting as trigger for focal lymphocytic thyroiditis.

As previously reported, “chronic lymphocytic thyroiditis frequently coexists with papillary thyroid carcinoma, positive TgAb and more favorable outcome” [21,47]. Souza et al. hypothesized that “it is possible that concurrent thyroiditis influences the homeostasis of PTC immune microenvironment and the autoimmune activity against the thyroid gland may exert a protective role in patients with PTC” [48].

In keeping with the previous literature [3,4], we think that PLNF represent very early dysplastic changes associated with an altered, immunogenic colloid substance. The presence of inflammatory lymphocytic infiltrate closely associated with the follicles with PLNF could represent the manifestation of the host response to these changes. In this early phase, the presence of an immune response could have a protective role, more effective if it consists of CD4+ and CD8+ T-effector active lymphocytes.

We agree that lymphocytic infiltrates play a main protective role against early dysplastic changes in the thyroid gland, as PLNF can be considered. On the contrary TgAb, as previously reported, “do not bind the complement molecule and are not cytotoxic” [49,50], in keeping with the unfavorable diagnostic and prognostic meaning of TgAb reported about thyroid cancer. Thus, TgAb positivity may indicate an early stage of the shift from cell-mediated immunity towards humoral immunity, less effective in fighting cancer.

This hypothesis needs further studies focusing on the complex composition of the inflammatory microenvironment, to assess whether immunological escape has been already triggered. This will be the purpose of our next studies.

We are aware that the main limitation of our study is represented by the low number of cases, but nevertheless our findings are promising and provide the basis for a subsequent study on a wider casuistry, including cases of differentiated papillary carcinoma and focusing on the complex composition of the inflammatory microenvironment, to assess whether immunological escape has been already triggered.

Finally, our findings can be translated to the diagnostical practice of FNAC of thyroid lesions. In the cytological assessment of thyroid nodules that in clinical practice might be classified as “indeterminate”, due to the presence of PLNF, the serum positivity of TgAb could recommend a closer clinical surveillance.

## 5. Conclusions

The present study shows that the presence of serum positivity for TgAb in surgically resected non-neoplastic thyroid glands is significantly related to the presence of PLNF and inflammatory lymphocytic infiltrate. On the contrary, no relation between serum TPOAb and the above-mentioned features was observed.

The presence of lymphocytic infiltrate in thyroid tissue could represent a defense mechanism, probably triggered by an incorrectly glycosylated Tg contained in the HBME1-positive colloid of follicles with early dysplastic changes. To our knowledge, the relation between serum TgAb, serum TPOAb, the presence of lymphocytic infiltrate, and of PLNF had not been investigated before and the findings of our study provide the bases for further investigation to well-define the early stage of thyroid carcinogenesis.

## Figures and Tables

**Table 1:** Distribution of thyroid diseases according to the presence of serum antibodies (TgAb+/TPOAb−; TgAb+/TPOAb+; TgAb−/TPOAb−; TgAb−/TPOAb+).

	NIFTP	Follicular Adenoma	Multi- Nodular Goiter	Graves Disease	Chronic Lymphocytic Thyroiditis
TgAb+/TPOAb- (7)	0	1	4	1	1
TgAb+/TPOAb+ (19)	2	2	6	1	8
TgAb−/TPOAb- (38)	0	7	30	0	1
TgAb−/TPOAb+ (6)	0	0	4	0	2
Total (70)	2	10	44	2	12

**Table 2:** Presence of “papillary-like” nuclear features (PLNF), lymphocytic infiltrates (LI), or both according to the presence of serum TgAb; Yates’s Chi-square: Statistical significance assumed for *p*-values < 0.05. OR = Odds Ratio.

	Cases	PLNF Present	PLNF Absent	LI Present	LI Absent	PLNF/LI Present	PLNF/LI Absent
TgAb+	26	23	3	23	3	21	1
TgAb−	44	16	28	14	30	13	27
	Tot. 70	*p* = 0.0001 OR: 13.42		*p* < 0.0001 OR: 15.71		*p* < 0.0001 OR: 43.62	

**Table 3:** Presence of PLNF, lymphocytic infiltrates (LI), or both according to the presence of serum TgAb and/or TPOAb; Yates’s Chi-square and Fisher’s exact test: Statistical significance assumed for *p*-values < 0.05. (n.s. = not significant).

	PLNF Present	PLNF Absent	LI Present	LI Absent
TgAb+/TPOAb+ (19)	18	1	17	2
TgAb+/TPOAb− (7)	6	1	5	2
Fisher’s exact test	*p* = 0.4738 (n.s)		*p* = 0.287 (n.s)	
TgAb−/TPOAb+ (6)	4	2	4	2
TgAb−/TPOAb− (38)	16	22	10	28
Yates’s Chi-square	*p* = 0.49 (n.s) OR: 3.5		*p* = 0.1218 (n.s) OR: 5.8	

## Data Availability

The data presented in this study are available on request from the corresponding author.
